# CRISPRi enables fast growth followed by stable aerobic pyruvate formation in *Escherichia coli* without auxotrophy

**DOI:** 10.1002/elsc.202100021

**Published:** 2021-11-30

**Authors:** Martin Ziegler, Lorena Hägele, Teresa Gäbele, Ralf Takors

**Affiliations:** ^1^ Institute of Biochemical Engineering University of Stuttgart Stuttgart Germany

**Keywords:** CRISPRi, fermentation, metabolic engineering, nitrogen limitation, pyruvate

## Abstract

CRISPR interference (CRISPRi) was applied to enable the aerobic production of pyruvate in *Escherichia coli* MG1655 under glucose excess conditions by targeting the promoter regions of *aceE* or *pdhR*. Knockdown strains were cultivated in aerobic shaking flasks and the influence of inducer concentration and different sgRNA binding sites on the production of pyruvate was measured. Targeting the promoter regions of *aceE* or *pdhR* triggered pyruvate production during the exponential phase and reduced expression of *aceE*. In lab‐scale bioreactor fermentations, an *aceE* silenced strain successfully produced pyruvate under fully aerobic conditions during the exponential phase, but loss of productivity occurred during a subsequent nitrogen‐limited phase. Targeting the promoter region of *pdhR* enabled pyruvate production during the growth phase of cultivations, and a continued low‐level accumulation during the nitrogen‐limited production phase. Combinatorial targeting of the promoter regions of both *aceE* and *pdhR* in *E. coli* MG1655 pdCas9 psgRNA_aceE_234_pdhR_329 resulted in the stable aerobic production of pyruvate with non‐growing cells at Y_P/S_  =  0.36 ± 0.029 g_Pyruvate_/g_Glucose_ in lab‐scale bioreactors throughout an extended nitrogen‐limited production phase.

**Abbreviations**
Atc: anhydrotetracycline
CRISPR: clustered regularly interspaced short palindromic repeats
CRISPRi: CRISPR interference
*E. coli*
: *Escherichia coli*
sgRNA: single‐guide RNA
TCA: tricarboxylic acid

## 1 INTRODUCTION

Pyruvate is a small metabolite at the intersection of glycolysis and the tricarboxylic acid (TCA) cycle. The production of pyruvate by biotechnical means is well established, for example, by Toray Industries using *Torulopsis glabrata* [1, 2]. Processes with high titer, yield and productivity have been described not only for *T. glabrata* but also for *E. coli* [3, 4, 5] and the economic feasibility of *E. coli* as a pyruvic acid producer from glucose has been examined in a case study [6]. However, applications of pyruvate are relatively few compared to other small organic acids: Pyruvate primarily serves as an additive for the synthesis of small‐volume specialty chemicals or pharmaceuticals such as L‐DOPA and is sold as a food additive [7, 8]. The importance of pyruvate for biotechnological applications therefore originates from its position in the central carbon metabolism serving as a precursor for other small molecule compounds. Established bioproducts derived from pyruvate include the amino acids alanine, isoleucine, leucine and valine [9], small carbon molecules such as lactate or isobutanol [10] and isoprenoids synthesized by the methylerythritol‐4‐phosphate (MEP) pathway [11]. A comprehensive analysis of the metabolic capabilities of *E. coli* for the production of non‐natural commercial products found 54 products available within only five reaction steps from pyruvate [12]. In particular, the production of isoprenoids in *E. coli* by the native MEP pathway has received considerable attention in the past years [13, 14]. There is thus renewed interest in the construction of microbial strains capable of accumulating pyruvate [15, 16, 17].

An important factor in engineering the productivity of heterologous pathways branching from pyruvate is the balance of precursor availability for growth and production. Pyruvate is usually produced from glucose and gene deletions or mutations with high impact on the catalytic activity of the pyruvate dehydrogenase complex result in acetate auxotrophy in aerobic conditions [18]. Whereas this enables decoupling of growth and pyruvate production, it also implies that any demand for reducing power and ATP exceeding the supply provided from glycolysis must be met by the co‐consumption of acetate which increases process cost and complexity [6]. Several studies have thus explored the possibility of throttling the flux from pyruvate to acetyl‐CoA to trigger the accumulation of pyruvate while maintaining a low level of flux through the TCA cycle to avoid acetate auxotrophy. The regulation of pyruvate dehydrogenase complex activity could be achieved by point mutations modulating catalytic activity [16, 17]. Alternatively, gene expression was controlled by promoter engineering [9] or by regulated expression of antisense RNA [19]. Strains accumulating pyruvate while maintaining an acceptable growth phenotype have the potential to serve as chassis for pyruvate‐derived products.

In recent years, CRISPRi has emerged as a powerful tool for the targetable reduction of gene expression in *E. coli* (for reviews, see [20, 21, 22]). CRISPRi uses the exogeneous catalytically inactive protein dCas9 from *S. pyogenes* in conjunction with single‐guide RNAs (sgRNA) to interfere with transcription of the target gene through steric hindrance [23, 24]. A sgRNA forms a stable complex with dCas9 which can then bind to a DNA region with complementarity to the targeting sequence and a protospacer adjacent motif (PAM). If the complex targets a region close to the transcription start of a single gene or operon, it may prevent binding of RNA polymerase which effectively represses the expression of the downstream genes. The repression strength in CRISPRi varies over several orders of magnitude depending on the distance of the binding site to the transcription start, the target strand, and mismatches in the targeting sgRNA [24, 25]. CRISPRi has already been applied successfully to improve the microbial production of a plethora of small compounds [20, 26‐28]. The technique is of particular interest for metabolic engineers because it facilitates the downregulation of essential genes which is helpful to find an optimal balance between competing processes such as growth and production [29]. Limitations of the technique mainly arise from potential off‐target effects and limited multiplexing [30, 31].

**PRACTICAL APPLICATION**

Pyruvate is an important cellular precursor at the intersection of glycolysis and tricarboxylic acid cycle (TCA). Reduced pyruvate flux into the TCA improves its availability for the formation of pyruvate‐derived products such as terpenoids synthesized via the methylerythritol‐4‐phosphate (MEP) pathway in *E. coli*. In this study, balanced reduction of pyruvate dehydrogenase activity by CRISPR interference was explored to trigger the accumulation of pyruvate while maintaining robust cellular growth and avoiding acetate auxotrophy. We demonstrate the applicability of the approach in exemplary aerobic fermentations including an extended nitrogen‐limited production phase. The strategy has the potential to improve titer and carbon conversion in the biotechnical production of pyruvate‐derived products.

Fed‐batch processes are usually the standard fermentation mode in white biotechnology but complementary techniques such as the partial decoupling of growth and production phases or in situ product removal are actively investigated [32, 33]. Small carbon‐based molecules such as pyruvate require no nitrogen for product formation and nitrogen limitation is thus a simple method to achieve decoupling of growth and production [16, 17]. Nitrogen‐limited processes are particularly attractive as nitrogen is a major component of biomass, nitrogen sources such as ammonia are usually cheap and easy to measure and the interplay of glucose and nitrogen metabolism in industrial hosts such as *E. coli* and *S. cerevisiae* is well characterized [34, 35].

Based on previous works in the field, we hypothesized that CRISPRi with suitable sgRNAs should enable the balanced aerobic production of pyruvate in nitrogen‐limited conditions. To explore the possibilities of CRISPRi we designed sgRNAs for the silencing of *aceE* by targeting the promoters *aceEp* and *pdhRp*. The repression of *aceEp* alone enabled the production of pyruvate in aerobic pH‐controlled fermentations during the exponential growth phase. However, it was not sufficient to create a stable production phenotype throughout an extended nitrogen‐limited production phase. Repression of transcription from *pdhRp* overcame this limitation and allowed the production of pyruvate at very low rates during a nitrogen‐limited production phase. Simultaneous targeting of *aceE* and *pdhR* in strain *E. coli* MG1655 pdCas9 psgRNA_aceE_234_pdhR_329 enabled substantial improvements in the fermentation performance.

## 2 MATERIALS AND METHODS

### 2.1 Media and buffer solutions

2xTY medium was prepared by autoclaving 16 g/L tryptone, 10 g/L yeast extract, and 5 g/L NaCl dissolved in demineralized water. For agar plates 18 g/L agar‐agar were added prior to autoclavation. SOC medium was prepared as described previously [36]. All cultivations were performed at 37°C.

Minimal medium for shaking flask experiments consisted of 20 g/L glucose, 2.0 g/L NaH_2_PO_4_⋅2H_2_O, 5.2 g/L K_2_HPO_4_, 4.56 g/L (NH_4_)_2_SO_4_, 15 g/L 3‐(*N*‐morpholino)propanesulfonic acid (MOPS) and 0.4% (V/V) trace elements stock solution. This medium was also used for microbioreactor cultivations and their precultures.

N‐lim minimal medium for precultures of bioreactor experiments consisted of 10 g/L glucose, 1.0 g/L NaH_2_PO_4_⋅2H_2_O, 2.6 g/L K_2_HPO_4_, 2.2 g/L (NH_4_)_2_SO_4_, 15 g/L 3‐(*N*‐morpholino) propanesulfonic acid (MOPS) and 0.2% (V/V) trace elements stock solution. N‐lim minimal medium for bioreactor experiments consisted of 70 g/L glucose, 1.0 g/L NaH_2_PO_4_⋅2H_2_O, 2.6 g/L K_2_HPO_4_, 2.2 g/L (NH_4_)_2_SO_4_ and 0.2 % (V/V) trace elements stock solution.

If strains with antibiotic resistance markers were cultivated in any liquid media or on 2xTY agar plates, appropriate antibiotics were added to media in the following concentrations: Chloramphenicol 25 μg/mL, disodium Carbenicillin 100 μg/mL. If necessary, inducers were added to minimal media in the following concentrations unless stated otherwise: Isopropyl β‐d‐1‐thiogalactopyranoside (IPTG) 1 mM, Anhydrotetracycline (Atc) 0.1 μg/mL.

The composition of trace element stock solution was 4.175 g/L FeCl_3_⋅6H_2_O, 0.045 g/L ZnSO_4_⋅7H_2_O, 0.025 g/L MnSO_4_⋅H_2_O, 0.4 g/L CuSO_4_⋅5H_2_O, 0.045 g/L CoCl_2_⋅6H_2_O, 2.2 g/L CaCl_2_⋅2H_2_O, 50 g/L MgSO_4_⋅7H_2_O and 55 g/L sodium citrate dehydrate. Stock solutions of salts, trace elements and sugars were autoclaved separately, and stock solutions of antibiotics were filter sterilized and stored at ‐20°C. All compounds were combined just before the experiments to prevent potential aging of media.

### 2.2 Bacterial strains and cloning of plasmids for CRISPR interference

All strains used in this study are listed in Table 1 and all primers used in this study are listed in Table 2.

**TABLE 1:** Strains used in this study

Strains	Strain Information/CRISPRi targets	Experimental series	Reference
*Escherichia coli* DH5α λ *pir*	Cloning strain		[16]
*E. coli* MG1655	Wild‐type strain		[16]
*E. coli* Top10 pdCas9	Contains dCas9 inducible by anhydrotetracycline		[24]^a^
*E. coli* Top10 pgRNA‐bacteria	Empty guideRNA plasmid		[24]^b^
*E. coli* MG1655 psgRNA_lacZ_236 pdCas9	*lacZ* (TTGGGAAGGGCGATCGGTGC)		[24]^c^
*E. coli* MG1655 psgRNA_lacZ_237 pdCas9	*lacZ* (GGCCAGTGAATCCGTAATCA)		This study
*E. coli* MG1655 psgRNA_lacZ_238 pdCas9	*lacZ* (AAGCATAAAGTGTAAAGCCT)		This study
*E. coli* MG1655 psgRNA_lacZ_239 pdCas9	*lacZ* (AGCGGATAACAATTTCACAC)		This study
*E. coli* MG1655 psgRNA_neg_241 pdCas9	‐		This study
*E. coli* MG1655 psgRNA_aceE_232 pdCas9	*aceE* (ACCTGTCTTATTGAGCTTTC)	1	This study
*E. coli* MG1655 psgRNA_aceE_233 pdCas9	*aceE* (CTGTCCCATTGAACTCTCGC)	1	This study
*E. coli* MG1655 psgRNA_aceE_234 pdCas9	*aceE* (TCTAATAACGTTGAGTTTTC)	1	This study
*E. coli* MG1655 psgRNA_aceE_235 pdCas9	*aceE* (AGCCAGTCGCGAGTTTCGAT)	1	This study
*E. coli* MG1655 psgRNA_aceE_232_aceE_234 pdCas9	*aceE* (ACCTGTCTTATTGAGCTTTC, TCTAATAACGTTGAGTTTTC)	2	This study
*E. coli* MG1655 psgRNA_aceE_232_aceE_235 pdCas9	*aceE* (ACCTGTCTTATTGAGCTTTC, AGCCAGTCGCGAGTTTCGAT)	2	This study
*E. coli* MG1655 psgRNA_aceE_233_aceE_234 pdCas9	*aceE* (CTGTCCCATTGAACTCTCGC, TCTAATAACGTTGAGTTTTC)	2	This study
*E. coli* MG1655 psgRNA_aceE_233_aceE_235 pdCas9	*aceE* (CTGTCCCATTGAACTCTCGC, AGCCAGTCGCGAGTTTCGAT)	2	This study
*E. coli* MG1655 psgRNA_aceE_233_pdhR_327 pdCas9	*aceE + pdhR* (CTGTCCCATTGAACTCTCGC, TCAAAACCTGTATGGACATA)	3	This study
*E. coli* MG1655 psgRNA_aceE_233_pdhR_328 pdCas9	*aceE + pdhR* (CTGTCCCATTGAACTCTCGC, TATTCACCTTATGTCCATAC)	3	This study
*E. coli* MG1655 psgRNA_aceE_233_pdhR_329 pdCas9	*aceE + pdhR* (TCTAATAACGTTGAGTTTTC, AGCCACTTGCCGAAGTCAAT)	3	This study
*E. coli* MG1655 psgRNA_aceE_234_pdhR_327 pdCas9	*aceE + pdhR* (TCTAATAACGTTGAGTTTTC, TCAAAACCTGTATGGACATA)	3	This study
*E. coli* MG1655 psgRNA_aceE_234_pdhR_328 pdCas9	*aceE + pdhR* (TCTAATAACGTTGAGTTTTC, TATTCACCTTATGTCCATAC)	3	This study
*E. coli* MG1655 psgRNA_aceE_234_pdhR_329 pdCas9	*aceE + pdhR* (TCTAATAACGTTGAGTTTTC, AGCCACTTGCCGAAGTCAAT)	3	This study
*E. coli* MG1655 psgRNA_pdhR_327 pdCas9	*pdhR* (TCAAAACCTGTATGGACATA)	4	This study
*E. coli* MG1655 psgRNA_pdhR_328 pdCas9	*pdhR* (TATTCACCTTATGTCCATAC)	4	This study
*E. coli* MG1655 psgRNA_pdhR_329 pdCas9	*pdhR* (AGCCACTTGCCGAAGTCAAT)	4	This study

^a^ pdCas9‐bacteria was a gift from Stanley Qi (Addgene plasmid # 44249; http://n2t.net/addgene:44249; RRID: Addgene_44249).

^b^ pgRNA‐bacteria was a gift from Stanley Qi (Addgene plasmid # 44251; http://n2t.net/addgene:44251; RRID: Addgene_44251).

^c^ Strain was constructed in this study according to information provided in the given reference.

**TABLE 2:** Primers used in this study

No.	Primer name	Sequence 5′ → 3′ (binding sequence)	Function
236	lacZ_236	TTGGGAAGGGCGATCGGTGCGTTTTAGAGCTAGAAAT GCAAGTTAAAATAAGGC	Fwd primer for iPCR [24]
237	lacZ_237	GGCCAGTGAATCCGTAATCAGTTTTAGAGCTAGAAATA CAAGTTAAAATAAGGC	Fwd primer for iPCR
238	lacZ_238	AAGCATAAAGTGTAAAGCCTGTTTTAGAGCTAGAAATA CAAGTTAAAATAAGGC	Fwd primer for iPCR
239	lacZ_239	AGCGGATAACAATTTCACACGTTTTAGAGCTAGAAATA CAAGTTAAAATAAGGC	Fwd primer for iPCR
232	aceE_232	ACCTGTCTTATTGAGCTTTCGTTTTAGAGCTAGAAATAG AAGTTAAAATAAGGC	Fwd primer for iPCR
233	aceE_233	CTGTCCCATTGAACTCTCGCGTTTTAGAGCTAGAAATAG AAGTTAAAATAAGGC	Fwd primer for iPCR
234	aceE_234	TCTAATAACGTTGAGTTTTCGTTTTAGAGCTAGAAATAG CAAGTTAAAATAAGGC	Fwd primer for iPCR
235	aceE_235	AGCCAGTCGCGAGTTTCGATGTTTTAGAGCTAGAAATA CAAGTTAAAATAAGGC	Fwd primer for iPCR
327	pdhR_327	TCAAAACCTGTATGGACATAGTTTTAGAGCTAGAAATA CAAGTTAAAATAAGGC	Fwd primer for iPCR
328	pdhR_328	TATTCACCTTATGTCCATACGTTTTAGAGCTAGAAATAG AAGTTAAAATAAGGC	Fwd primer for iPCR
329	pdhR_329	AGCCACTTGCCGAAGTCAATGTTTTAGAGCTAGAAATA CAAGTTAAAATAAGGC	Fwd primer for iPCR
240	sgRNA_r	ACTAGTATTATACCTAGGACTGAGCTAGC	Rev primer for iPCR [25]
241	sgRNA_neg	GTTTTAGAGCTAGAAATAGCAAGTTAAAATAAGGC	Fwd primer for iPCR [25]
242	sgRNA_seq_f	GGGTTATTGTCTCATGAGCGGATACATATTTG	Sequencing of psgRNA [25]
452	aceE_forward	GTCACAGCCACATTCAGTC	Fwd primer for qPCR
453	aceE_reverse	TACCTTCCTCAGCACCTTC	Rev primer for qPCR
454	mdoG_forward	TCGATACCCCGGTCAAAATA	Fwd primer for qPCR [37]
455	mdoG_reverse	CGGGCTGTATTTGATTCGTT	Rev primer for qPCR [37]

Cloning of psgRNA plasmids was conducted as described previously [38]. Briefly, primers were 5′ phosphorylated using T4 polynucleotide kinase. Next, pgRNA‐bacteria or a psgRNA plasmid was amplified by inverse PCR (iPCR) using a reverse primer binding the plasmid in the promoter region of the sgRNA expression cassette and a forward primer containing the complementary 20 nucleotide target binding sequence for CRISPR interference to be introduced flanked by an annealing region to the plasmid. After purification of the PCR reaction, DpnI degradation of the plasmid template, and separation of products on an agarose gel, bands at 2.6 kb were extracted and the purified DNA fragments circularized by blunt‐end ligation using T4 DNA ligase. *E. coli* DH5α λ *pir* was transformed with 2 μL of the ligation reaction by electroporation and regenerated in SOC medium. Cells were then plated on 2xTY agar plates and incubated at 37°C overnight. Cells from a single colony were grown in 2xTY, the plasmids extracted using E.Z.N.A. Plasmid DNA mini Kit I (omega BIO‐TEK) according to the manufacturer's instructions and the insert coding for the sgRNA verified by sequencing.

Cloning of psgRNA plasmids containing more than one sgRNA was performed using iPCR and BioBrick assembly cloning as described elsewhere [25, 39]. In short, the donor sgRNA plasmid was digested using EcoRI and BamHI and the recipient plasmid digested with EcoRI and BGlII. Fragments were separated on agarose gels, extracted, purified, and ligated using T4 DNA ligase. *E. coli* DH5α λ *pir* was transformed with 5 μL of the ligation reaction by electroporation and regenerated in SOC medium. Cells were then plated on 2xTY agar plates and incubated at 37°C overnight. Cells from a single colony were grown in 2xTY, the plasmids extracted using E.Z.N.A. Plasmid DNA mini Kit I (omega BIO‐TEK) according to the manufacturer's instructions and the insert coding for the sgRNA verified by sequencing.

To construct the actual production strains, *E. coli* MG1655 was transformed with 1 μL of purified psgRNA plasmid by electroporation, regenerated in SOC medium, plated on 2xTY agar plates, and incubated at 37°C overnight. Electrocompetent cells were prepared from a single colony and transformed with 5 μL of pdCas9 using identical procedures. The resulting strains carrying two plasmids were grown in 2xTY and stored as glycerol stocks at ‐70°C.

### 2.3 ẞ‐Galactosidase assay

The activity of ẞ‐galactosidase, the product of *lacZ*, was assayed according to Jeffrey Miller's protocol with minor adaptions [40]. Baffled 100 mL shaking flasks containing 10 mL 2xTY medium were inoculated with a single colony from an agar plate streak and incubated with appropriate antibiotics at 37°C and 130 rpm. After 30 min, isopropyl‐*β*‐D‐thiogalactopyranosid (IPTG) was added to a final concentration of 1 mM. Cells were grown to mid‐log phase and the optical density at 600 nm measured. A total of 2 mL of biosuspension were harvested by centrifugation for 2 min at 12,000 *g*, the supernatant discarded, and the cell pellet resuspended in 2 mL of Z‐buffer (60 mM Na_2_HPO_4_⋅2H_2_O, 40 mM NaH_2_PO_4_⋅H_2_O, 10 mM KCl, 1 mM MgSO_4_, 50 mM ẞ‐mercaptoethanol, pH adjusted to 7.0 with NaOH/H_3_PO_4_ prior to addition of ẞ‐mercaptoethanol). An appropriate volume of resuspended cell suspension was further diluted in Z‐buffer to yield 1 mL of assay sample solution. A total of 1  mL of diluted cells were lysed with 50 μL of chloroform and 25 μL of 0.1% sodium dodecyl sulfate (SDS) solution. After incubation for 5 min, 200 μL of substrate solution (4 g/L o‐nitrophenyl‐ẞ‐D‐galactopyranoside (OPNG) dissolved in Z‐buffer) were added and the time until the sample turned yellow was recorded. The reaction was stopped by adding 500 μL stop solution (1 M Na_2_CO_3_ in deionized water) and the samples centrifuged for 7 min at 12000 *g*. The supernatant was transferred into PMMA semi‐micro cuvettes and the absorption at 420 nm was measured. Miller units were calculated according to the following equation, where t is the time of reaction in minutes and V the volume of cell suspension used to correct for dilution of samples in Z‐buffer:

$$\begin{array}{ccc} & & \beta -galactosidase\;activity\left[miller\;units\right]\\ & & \;=\left(OD_{420}\times 1000\right)/\left(OD_{600}\times t\cdot \times \cdot V\right) \end{array}$$

### 2.4 Shaking flask cultivations

Strains were streaked from glycerol stock cultures on 2xTY agar plates and grown overnight at 37°C. For precultures, a 100 mL baffled shaking flask containing 20 mL minimal medium was inoculated with a single colony and incubated at 37°C on a rotary shaker set to 130 rpm for 16 ‐ 40 h. For main cultures, a 500 mL baffled shaking flask containing 55 mL of minimal medium was inoculated with preculture to a starting OD of 0.2 and cultivated at 37°C on a rotary shaker set to 130 rpm.

### 2.5 Bioreactor cultivations

Precultures for bioreactor experiments were inoculated from glycerol stock cultures by transferring 333  μL of glycerol stock culture into a 100 mL baffled shaking flask containing 20 mL N‐lim minimal medium. Precultures were incubated at 37°C on a rotary shaker set to 130 rpm overnight. On the next morning, a glass bioreactor containing 200 mL of N‐lim minimal medium was inoculated with preculture to a starting OD of 0.2. Glass bioreactors were equipped with a temperature control set to 37°C and magnetic stirrers set to 500 rpm. Throughout the cultivation stirring speed and gassing were kept constant at 500 rpm and 300 mL/min. DO tension was monitored and never dropped below 30% saturation to ambient air partial oxygen pressure. The pH was kept constant at 7.0 by automated addition of 3 M NaOH. Prior to fermentation start a single droplet (about 10 μL) of Struktol J647 antifoaming agent was added to the vessel to prevent potential foaming.

### 2.6 Microbioreactor cultivations and automated sampling

Strains were streaked from glycerol stock cultures on 2xTY agar plates and grown overnight at 37°C. A single colony was picked to inoculate precultures in 5 mL tubes in 2xTY medium which were incubated at 37°C on a rotary shaker set to 130 rpm for 6.5 h. A total of 100 mL baffled shaking flasks containing 20 mL minimal medium were inoculated to a starting OD of 0.04 and cultivated at 37°C on a rotary shaker set to 130 rpm overnight. The main cultivation was conducted in a microbioreactor system (BioLector, m2p labs, Baesweiler, Germany) equipped with microplates (FlowerPlate, MTP‐48‐BOH1, m2p labs) with 1.1 mL minimal medium including anhydrotetracycline in each well. Wells were inoculated to an OD of 0.2 and cultivation ensued at 37°C with relative humidity of 85% and shaking set to 1100 rpm. Sealing foil for automated cultivations (F‐GPRS48‐10) was used to reduce evaporation and enable automated sampling. Optical density (via backscatter), dissolved oxygen concentration and pH were measured every 5 min. Sampling was carried out automatically with the liquid handing platform of the BioLector system. Samples for gene expression analysis were taken at the backscatter value of 6.5 (corresponding to biomass concentration of around 0.5 g/l) with a block time of 0.1 h. 250 μL of freshly sampled biosuspension was directly transferred into 50 μL of RNA/DNA shield (Zymo Research, Freiburg, Germany) preloaded in a 96 well plate. Samples were then transferred into lysis tubes containing 700 μL lysis buffer (Zymo Research, Irvine, USA), flash frozen in liquid nitrogen and stored at ‐20°C until day of RNA isolation.

### 2.7 Analytical procedures

Bacterial growth was monitored by measurements of optical density at 600 nm. Biosuspension samples were appropriately diluted with 0.9% NaCl solution and cell dry weight calculated from these values assuming a correlation factor of 0.3 [16].

2 mL of freshly sampled biosuspension were centrifuged at 12,000 *g* for 2 min and aliquots of the resulting supernatant frozen until further analysis. Isocratic HPLC using a RI detector (1200Series, Agilent) with a Rezex ROA‐Organic acid H^+^ column (Phenomenex) for separation was used to measure glucose, acetic acid, lactate, 2‐oxoglutarate, ethanol, formate and succinate as described previously [16]. Glucose concentration was alternatively determined by D‐Glucose UV‐Test Kit (R‐Biopharm, Darmstadt, Germany) and acetic acid concentration by Acetic acid UV‐Test Kit (R‐Biopharm, Darmstadt, Germany). Ammonium concentration was determined by Ammonium cuvette test LCK 303 or LCK 304 (Hach Lange, Düsseldorf, Germany). Pyruvate was determined by an enzymatic assay measuring the consumption of NADH upon conversion of pyruvate to lactate by L‐lactate dehydrogenase (LDH). L‐lactate dehydrogenase suspension (L2500, Merck) was diluted 1:10 in 2.5 M (NH_4_)_2_SO_4_ solution. 500 μL of 100 mM tris (pH 7.4), 100 μL of 2 mM NADH and 290 μL deionized water were mixed in an acryl cuvette and 100 μL of appropriately diluted sample was added. The absorbance at 365 nm was measured and 10 μL of LDH suspension was added to initiate the reaction. After incubation for 10 min at room temperature the absorbance at 365 nm was measured again and the resulting difference in absorbance used to calculate the pyruvate content of the sample.

### 2.8 Gene expression analysis

RNA isolation was conducted using Quick RNase Mini Kit (Zymo Research, Irvine, USA) according to the manufacturer's instructions. Isolated RNA was DNAse treated with Turbo DNAse (Thermo Fisher Scientific, Walthan, USA) and concentrated with the RNA Clean and Concentrator 5 Kit (Zymo Research, Irvine, USA). For complementary DNA (cDNA) synthesis, 1 μg of isolated RNA was treated with Reverse Transcriptase Superscript IV (Thermo Fisher Scientific, Walthan, USA) according to the manufacturer's instruction using random hexamers (NEB, Ipswich, USA). After RT‐PCR 1 μL of RNaseH (NEB, Ipswich, USA) was added and samples were incubated at 37°C for 20 min to digest initial RNA. For quantitative PCR (qPCR), cDNA samples were diluted by a factor of 6 with ddH2O. qPCR master mix contained 7.5 μL 2x ORATM SEE qPCR Green ROX L Mix (highQu, Kraichtal, Germany), 0.4 μL forward primer, 0.4 μL reverse primer, and 4.7 μL ddH2O per reaction. Primers are given in Table 2. Samples were measured in technical triplicates. Controls included a non‐RT control and a non‐template control. A five‐step 1:10 dilution series of pooled cDNA was prepared for the determination of amplification efficiency. qPCR was conducted on a qTower^3^ (Analytik Jena, Jena, Germany): 95°C for 3 min, 40 cycles of 95°C for 5 s, 59°C for 15 s, 72°C for 15 s and a final ramp from 65 to 95°C (0.5°C steps every 5 s). Expression of *aceE* was standardized to the expression of *mdoG* [41] and included corrections for amplification efficiency [42]. One‐sided t‐tests (α = 0.05) including Bonferroni‐Holm correction for repeated testing [43] were used to test the differences between engineered strains and the wild‐type. A post‐hoc Games‐Howell test was calculated to determine differences between group means [44].

## 3 RESULTS

Our goal was to apply CRISPRi to throttle the flux from pyruvate to the TCA cycle. We aimed for a strain that accumulated pyruvate aerobically while maintaining an acceptable growth phenotype without acetate auxotrophy. The target phenotype can be obtained by balanced reduction of the activity of the pyruvate dehydrogenase complex [16, 17]. Therefore, our primary knockdown target was *aceE* which encodes a subunit of the pyruvate dehydrogenase complex. Additionally, we planned on using the strains in two‐phase fermentations with an initial growth phase and a subsequent nitrogen‐limited production phase. To achieve these goals, we created in total four series of knockdown strains each based on different silencing strategies. In the first series, single silencing of *aceE* was tested. Knockdown strains of the second series were subject to combinatorial silencing of *aceE*. The third series was engineered for simultaneous silencing of *aceE* and *pdhR*. For the fourth and final series, we tested single silencing of *pdhR*. Strains of all four series were tested in aerobic shaking flasks. One knockdown strain from the first, third, and fourth series each was characterized in lab‐scale reactors including a nitrogen‐limited production phase.

### 3.1 Identification of binding sites for CRISPR interference

For CRISPRi, we used the two‐plasmid system described by Qi et al. [24] employing pdCas9 with an anhydrotetracycline inducible dCas9 and psgRNA containing constitutively expressed sgRNA templates [24]. The crucial factor for gene silencing by CRISPRi is the design of sgRNAs. As the scope of our experiments was limited, we manually examined the DNA sequence around the transcription start site of promoters for suitable target sites and used BLAST to exclude candidates with potential off‐target effects based on sequence similarity. To verify this simplistic approach, we designed three sgRNAs targeting *lacZp* and conducted beta‐galactosidase assays to gain an estimate of repression efficiencies in induced or non‐induced state. All sgRNAs targeting *lacZp* led to strong reduction of ẞ‐galactosidase activity and thus were sufficient to knock‐down *lacZ* (S1).

**FIGURE 1:**
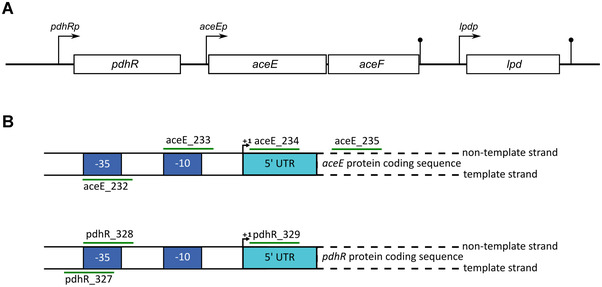
(A) Structure of the *pdhR*‐*aceEF*‐*lpd* operon. (B) sgRNA binding sites. Four binding sites (232, 233, 234 and 235) were chosen for CRISPRi targeting *aceE* and three binding sites (327,328 and 329) for CRISPRi against *pdhRp*

We then designed sgRNAs for the silencing of *aceE*. The *E. coli* gene *aceE* is part of the genomic *pdhR*‐*aceEF*‐*lpd* operon (Figure 1 A) which is primarily transcribed from *pdhRp* with minor contributions from the internal promoter *aceEp*, potentially involving σ^S^ [45, 46]. PdhR represses the entire operon and autoregulates its own synthesis by binding to the *pdhR* promoter region. Repression by PdhR is relieved by pyruvate and PdhR controls an additional small regulon of about 20 genes [47]. CRISPRi can inhibit transcription by blocking initiation or elongation. When inhibiting transcriptional elongation, targeting the non‐template strand is in general more effective than targeting the template strand [24]. We drafted two potential approaches: First, CRISPRi targeting *aceEp* should block both initiation from *aceEp* as well as hinder elongation of transcripts originating from *pdhRp*. Second, CRISPRi targeting *pdhRp* should effectively block initiation for the entire *pdhR*‐*aceEF*‐*lpd* operon while mimicking the regulatory effects of a *pdhR* deletion [48]. To explore diverse target sites, we chose three sites around *aceEp* (232, 233 and 234), one on the template‐strand and two on the non‐template strand, and an additional target site close to the start of the coding region of *aceE* (235) on the non‐template strand (Figure 1 B). We then identified three target sites around *pdhRp* (327, 328, and 329) which we deemed suitable for blocking transcriptional initiation from this promoter. We created four series of strains to test CRISPRi against *aceEp* in different constellations (Table 1). The sgRNAs were individually or sequentially cloned into psgRNA, and transformation of *E. coli* MG1655 with these plasmids and pdCas9 yielded *aceE* knockdown strains. In the first series only the promoter region of *aceE* (232, 233, 234, and 235) was targeted to avoid potential side‐effects of *pdhR* repression. The second series consisted of four different combinations of sgRNAs targeting *aceE* (232+234, 232+235, 233+234, and 233+235). The third series then aimed at combinatorial targeting of the promoter regions of both *aceE* and *pdhR* (233+327, 233+328, 233+329, 234+327, 234+328, and 234+329), and in the fourth series only the promoter region of *pdhR* (327, 328, and 329) was targeted.

### 3.2 Shaking flask fermentations of strains with *aceE* silencing

**FIGURE 2:**
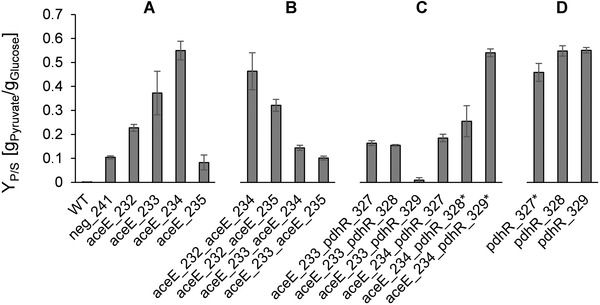
Pyruvate yield in shaking flask fermentations of *E. coli* MG1655 pdCas9 with different psgRNAs. Data is grouped into four series. Error bars indicate SEM (n = 3; *n = 2). (A) Wild‐type reference (no plasmids), empty psgRNA (neg_241), and first series, silencing of *aceE*. (B) Second series, combinatorial silencing of *aceE*. (C) Third series, simultaneous silencing of *aceE* and *pdhR*. (D) Fourth series, silencing of *pdhR*

**FIGURE 3:**
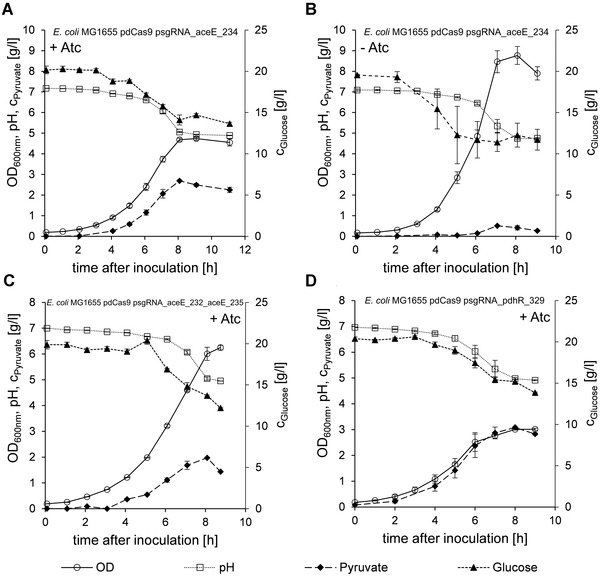
Shaking flask fermentations of knockdown strains. Error bars indicate SEM (n = 3). (A + B) *E. coli* pdCas9 psgRNA_aceE_234 with addition of the inducer anhydrotetracycline (A) or without anhydrotetracycline (B). (C) *E. coli* MG1655 pdCas9 psgRNA_aceE_232_aceE_235. (D) *E. coli* MG1655 pdCas9 psgRNA_pdhR_329

Strains from the four series were cultivated in aerobic shaking flasks in minimal medium with 0.1 μg/mL anhydrotetracycline. Over the course of the fermentations cell growth, the accumulation of pyruvate, the consumption of glucose, and the acidification of the medium were regularly measured. All sgRNAs individually triggered the accumulation of pyruvate in the first and fourth series of strains, but pyruvate yield from glucose varied considerably (Figure 2 A + D). Exemplary cultivation data from shaking flask experiments is shown in Figure 3.

Among the strains of the first series *E. coli* MG1655 pdCas9 psgRNA_aceE_234 showed the strongest pyruvate production (Figure 2 A and Figure 3 A). As we had observed leakiness of the expression system before (S1) we conducted shaking flask fermentations of this strain without addition of anhydrotetracycline and observed minor accumulation of pyruvate (Figure 3 B). To clarify the influence of anhydrotetracycline concentration on the silencing efficacy we chose a strain from the first series with intermediate pyruvate accumulation, *E. coli* MG1655 pdCas9 psgRNA_aceE_233, and cultivated it with varying anhydrotetracycline concentrations. The addition of as little as 0.01 μg/mL anhydrotetracycline was sufficient to induce the system and trigger the accumulation of pyruvate at the expense of biomass formation (S2). Concentrations up to 0.5 μg/mL of anhydrotetracycline were well tolerated, but at 1.0 μg/mL anhydrotetracycline growth inhibition without additional pyruvate production occurred. Small amounts of pyruvate were produced even in the absence of inducer. We concluded that the initially chosen 0.1 μg/mL anhydrotetracycline was well within the working range and continued to use this concentration.

As multiplex CRISPRi against a single gene or multiple genes was successfully applied in other studies to obtain desired phenotypes [49, 50] we initially hypothesized that combinatorial silencing in the second and third series should lead to stronger repression and higher pyruvate accumulation, but this was not generally the case (Figure 2 B+C). In fact, none of the strains from the second series showed beneficial properties surpassing those of the first series (Figure 2 A + B). Except for *E. coli* MG1655 pdCas9 psgRNA_aceE_232_aceE_235 (Figure 3 C) none of the combinatorial knockdown strains targeting the promoter region of *aceE* could reach the pyruvate yield of the respective single knockdown strains. On the contrary, the combinatorial silencing approach appeared to be detrimental in general. Similarly, in the third series only *E. coli* MG1655 pdCas9 psgRNA_aceE_234_pdhR_329 achieved a comparable pyruvate yield as the strains from the fourth series (Figure 2 D).

### 3.3 Expression of *aceE* in microbioreactor cultivations

**FIGURE 4:**
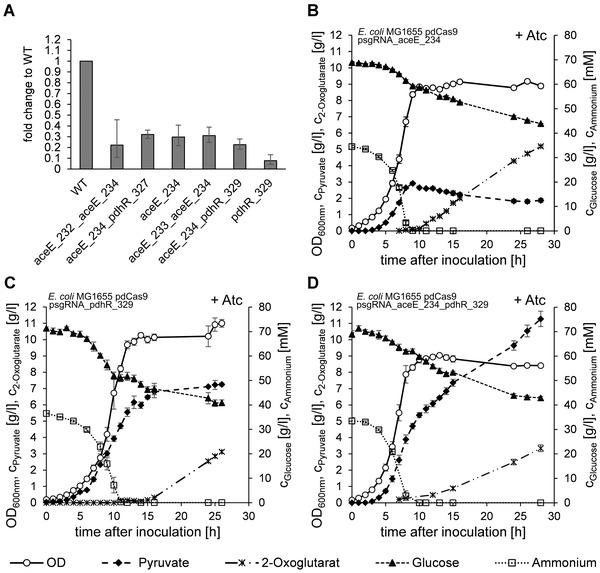
(A) Relative expression of *aceE* to the housekeeping gene *mdoG* in microbioreactor cultivations. Data is normalized to the expression of the wild‐type reference (no plasmids). Mean fold change and 95% confidence interval are shown. (B + C + D) Bioreactor cultivations of knockdown strains. The aerobic lab‐scale fermentations were carried out with excessive glucose. Depletion of ammonia initiates the nitrogen‐limited second process phase. Error bars indicate SEM. (B) *E. coli* MG1655 pdCas9 psgRNA_aceE_234 (n = 3). (C) *E. coli* MG1655 pdCas9 psgRNA_pdhR_329 (n = 3). (D) *E. coli* MG1655 pdCas9 psgRNA_aceE_234_pdhR_329 (n = 5)

In order to elucidate the varying levels of pyruvate accumulation observed in the shaking flask experiments we chose to cultivate a subset of the strains in microbioreactors and measured the expression of *aceE* in the exponential growth phase by qPCR (Figure 4 A). All investigated knockdown strains had significantly reduced expression of *aceE* compared to *E. coli* MG1655 (one‐tailed *t*‐tests including Bonferroni‐Holm correction, see S3) which confirms the functionality of CRISPRi in the strains. However, the differences in expression levels between the individual knockdown strains are small and cannot directly explain the varying pyruvate yield in the shaking flask experiments. A post‐hoc Games‐Howell test (α  =  0.05, see S3) indicated that among the different knockdown strains only *E. coli* MG1655 pdCas9 psgRNA_pdhR_329 had any significantly reduced expression which does not reflect the differences in pyruvate yield observed in the shaking flask experiments.

### 3.4 Bioreactor cultivations of *aceE* knockdown strains

One of our primary goals was to enable the production of pyruvate during an extended nitrogen‐limited production phase. We chose to characterize one promising strain each from the first, third and fourth series: *E. coli* MG1655 pdCas9 psgRNA_aceE_234, *E. coli* MG1655 pdCas9 psgRNA_aceE_234_pdhR_329, and *E. coli* MG1655 pdCas9 psgRNA_pdhR_329. Strains were cultivated in aerobic lab‐scale fermenters with controlled pH. The composition of the minimal medium included excessive glucose and trace elements, with ammonium as the limiting nutrient. All strains displayed comparable fermentation courses (Figure 4 B + C + D) but had remarkable differences in yields and rates (Table 3).

**TABLE 3:** Yield coefficients and fermentation parameters of bioreactor fermentations with extended nitrogen‐limited production phase

		*E. coli* MG1655 pdCas9 psgRNA_aceE_234		*E. coli* MG1655 pdCas9 psgRNA_pdhR_329		*E. coli* MG1655 pdCas9 psgRNA_aceE_234_pdhR_329	
Symbol	Unit	Exp. phase	N‐lim. phase	Exp. phase	N‐lim. phase	Exp. phase	N‐lim. phase
μ	[h^‐1^]	0.414 ± 0.0042^a^	‐	0.36 ± 0.010	‐	0.472 ± 0.0078	‐
Y_X/S_	[g_CDW_/g_Glucose_]	0.270 ± 0.0072	‐	0.10 ± 0.014	‐	0.29 ± 0.036	‐
q_s_	[g_Gluocse_/g_CDW_/h]	1.53 ± 0.046	0.30 ± 0.011	3.7 ± 0.44	0.22 ± 0.046	1.8 ± 0.24	0.38 ± 0.013
Y_P/S_	[g_Pyruvate_/g_Glucose_]	0.40 ± 0.018	‐	0.201 ± 0.0056	0.28 ± 0.082	0.50 ± 0.065	0.36 ± 0.029
q_p_	[g_Pyruvate_/g_CDW_/h]	0.61 ± 0.012	‐0.021 ± 0.0026	0.8 ± 0.11	0.056 ± 0.049	0.83 ± 0.023	0.135 ± 0.0095
Y_2‐Oxo/S_	[g_2‐Oxoglutarate_/g_Glucose_]	0.010 ± 0.0018	0.332 ± 0.0073	–	0.32 ± 0.068	0.0390 ± 0.0065	0.16 ± 0.012
q_2‐Oxo_	[g_2‐Oxoglutarate_/g_CDW_/h]	0.016 ± 0.0029	0.010 ± 0.0016	–	0.066 ± 0.0022	0.063 ± 0.0022	0.062 ± 0.0054

^a^ Errors indicate SEM, *E. coli* MG1655 pdCas9 psgRNA_aceE_234 (n = 3), *E. coli* MG1655 pdCas9 psgRNA_pdhR_329 (n = 3). *E. coli* MG1655 pdCas9 psgRNA_aceE_234_pdhR_329 (n = 5).

During the initial exponential batch‐phase *E. coli* MG1655 pdCas9 psgRNA_aceE_234_pdhR_329 grew faster than *E. coli* MG1655 pdCas9 psgRNA_aceE_234 and *E. coli* MG1655 pdCas9 psgRNA_pdhR_329. While *E. coli* MG1655 pdCas9 psgRNA_pdhR_329 grew slower than the other strains, its glucose consumption rate was remarkably elevated leading to overall low yields for biomass and pyruvate. All strains accumulated pyruvate during the growth phase, albeit at different yields (Table 3).

Upon depletion of ammonium and entry into the nitrogen‐limited phase the fermentation patterns of the strains increasingly diverged. All strains essentially ceased to grow, only minor increases or decreases in OD were measured. *E. coli* MG1655 pdCas9 psgRNA_aceE_234 stopped pyruvate production entirely, and instead slowly consumed pyruvate over the remaining course of the fermentation. In contrast, *E. coli* MG1655 pdCas9 psgRNA_pdhR_329 continued to accumulate pyruvate at a low rate throughout the nitrogen‐limited production phase to a final concentration of 7.25 g/L. The highest continued pyruvate production was observed for *E. coli* MG1655 pdCas9 psgRNA_aceE_234_pdhR_329, and a pyruvate content of 11.28 g/L was measured in the final fermentation samples. The primary byproduct for all strains was 2‐oxoglutarate which *E. coli* MG1655 pdCas9 psgRNA_aceE_234 produced to a final titer of 5.19 g/L (Figure 4 B). *E. coli* MG1655 pdCas9 psgRNA_aceE_234_pdhR_329 produced a total of 3.34 g/L 2‐oxoglutarate with lactate (0.40 g/L) and acetate (0.37 g/L) as minor byproducts. Minor byproduct formation was similar for the other strains (data not shown).

Comparing the performance of the strains, *E. coli* MG1655 pdCas9 psgRNA_aceE_234_pdhR_329 was clearly superior to the two single knockdown strains. Not only did it achieve the stable production of pyruvate during the nitrogen‐limited phase with significantly higher biomass specific productivity (pairwise two‐tailed *t*‐tests, *p* < 0.01 each) but also had significantly higher maximum specific growth rate than the two other strains during the exponential phase (pairwise two‐tailed *t*‐tests, *p* < 0.01 each). Probably owing to constant pyruvate production it also had a significantly higher specific glucose consumption rate during the nitrogen limited phase (pairwise two‐tailed *t*‐test, *p* < 0.01). The production characteristics of *E. coli* MG1655 pdCas9 psgRNA_pdhR_329 resembled an intermediate phenotype between *E. coli* MG1655 pdCas9 psgRNA_aceE_234 and *E. coli* MG1655 pdCas9 psgRNA_aceE_234_pdhR_329 during the nitrogen‐limited phase. The strain might thus be useful to avoid the excess production of pyruvate if a metabolic production pathway with comparably low flux such as the MEP pathway is used [13].

## 4 DISCUSSION

CRISPRi enables the rapid targeted silencing of virtually any non‐essential gene for the purpose of metabolic engineering. In this study, we applied CRISPRi to reduce the expression of *aceE* resulting in the accumulation of pyruvate in aerobic fermentations. All sgRNAs tested enabled pyruvate production in shaking flasks, but at substantially differing yields. qPCR measurements of *aceE* expression for selected strains confirmed the functionality of the used CRISPRi system on transcript level. The simultaneous targeting of *aceEp* and *pdhRp* in *E. coli* MG1655 pdCas9 psgRNA_aceE_234_pdhR_329 lead to the stable production of pyruvate at low metabolic rates during a nitrogen‐limited production phase.

The sgRNAs targeting *aceE* were designed to block transcript elongation originating from *pdhRp* and initiation from *aceEp*. Given that construct psgRNA_aceE_235, designed to only block elongation, showed the poorest performance we conclude that effective silencing is most easily accomplished by targeting promoter regions. Construct psgRNA_aceE_232 was clearly less effective than psgRNA_aceE_233 and psgRNA_aceE_234 confirming the importance of targeting the non‐template strand. In the case of *pdhRp* all target sites were located close to the transcription initiation site and appeared well suited. These observations are well in line with previously published findings concerning the choice of CRISPRi targets [24]. Besides the good silencing efficacy, we observed substantial repression in the absence of inducer and even the lowest tested anhydrotetracycline concentration of 0.01 μg/mL was sufficient to exert repression (S2). We conclude that silencing efficacy should be fine‐tuned by sgRNA design rather than by expression level of dCas9 if a specific level of activity is desired. Low cellular dCas9 levels are in principle desirable anyway, as toxic effects can occur if sgRNAs with certain seed sequences are used in conjunction with high dCas9 concentration [51].

Combinatorial CRISPRi in the second series of strains against multiple target sites in *aceEp* was largely unsuccessful. While gene expression measurements (Figure 4 A) did not indicate a negative impact of combinatorial silencing on the repression efficiency, there were detrimental effects on the phenotypic level. Except for *E. coli* MG1655 pdCas9 psgRNA_aceE_232_aceE_235 we could not achieve relevant improvements in any of the *aceE* double knockdown strains. Targeting the coding region of a gene with multiple sgRNAs was successful in some other studies if the two target sites were sufficiently apart [24, 50]. Data collected with Cas9 nickase showed that an offset of 8 base pairs was sufficient to allow the binding of multiple Cas9 complexes [52]. This condition was fulfilled by all double knockdown strains except *E. coli* MG1655 pdCas9 psgRNA_aceE_233_aceE_234. The inefficacy of most silencing combinations in our experiments could potentially arise from the expression of multiple competing sgRNAs [30, 31]. Only recently, a strategy to couple dCas9 expression to the binding strength of multiple sgRNAs was proposed to solve such issues [53].

The stable production of pyruvate during the nitrogen‐limited production phase in the bioreactor fermentations was only possible by targeting *pdhRp*. Pyruvate accumulation and the repression of *pdhR* can potentially influence cellular regulatory cascades. For example, deletions of *pdhR* lead to overexpression of the pyruvate dehydrogenase complex and reduced maximum specific growth rates [54]. In wild‐type *E. coli* PdhR autoregulates its own synthesis and serves as a regulator to 16‐23 other genes [47]. Its central function is to relieve the repression of *pdhRp* at high pyruvate concentrations, thereby enhancing the expression of *pdhR*, *aceE*, and *aceF* which accelerates pyruvate degradation to acetyl‐CoA. Moreover, the PyrSR and BtsSR systems also sense pyruvate and each alters the expression of a small set of regulated genes [55, 56]. Despite the repression of *pdhR* in several knockdown strains and the concomitantly high pyruvate concentration, we did not observe detrimental effects other than reduced maximum growth rates in any strain. Given the binary outer circumstances – glucose excess and complete nitrogen starvation – during the nitrogen‐limited phase in the bioreactor fermentations of *E. coli* MG1655 pdCas9 psgRNA_aceE_234_pdhR_329 we presume that other regulatory responses such as the Ntr regulon dominated cellular adaptation. On the level of metabolite control, the concentration of 2‐oxoglutarate controls glucose uptake by competition with phosphoenolpyruvate for its binding site at the phosphotransferase system and limits the metabolic rates in prolonged nitrogen starvation [35]. Glucose uptake was strongly reduced during the extended production phase compared to the exponential phase in all bioreactor fermentations. However, the continued accumulation of 2‐oxoglutarate during the nitrogen‐limited phase did not further decrease the specific glucose uptake rates of the individual strains. Glucose uptake was thus either enabled by continued 2‐oxoglutarate export or through the activation of other mechanisms. In another study, glucose uptake reduction in nitrogen‐limited conditions was alleviated by moderate overexpression of *ptsI* which indicates that the cellular levels of 2‐oxoglutarate, PtsI and phosphoenolpyruvate are naturally tightly balanced [57].

Even though *E. coli* MG1655 pdCas9 psgRNA_aceE_234_pdhR_329 and *E. coli* MG1655 pdCas9 psgRNA_pdhR_329 accumulated less 2‐oxoglutarate than *E. coli* MG1655 pdCas9 psgRNA_aceE_234 during the nitrogen‐limited production phase, a substantial flux into the TCA cycle remained. Additional repression of pyruvate dehydrogenase activity and subsequently increased pyruvate yield from glucose could potentially be achieved by CRISPRi targeting the coding sequence of *aceF* and adjusting dCas9 levels for optimal repression [53]. Deletions or repression of *poxB* and *ldhA* would likely further increase pyruvate yield from glucose and reduce the accumulation of lactate. Since the experimental yields observed during the fermentations of *E. coli* MG1655 pdCas9 psgRNA_aceE_234_pdhR_329 were lower in the nitrogen‐limited fermentation phase, pyruvate yield also appeared to be dependent on the growth rate or the specific glucose uptake rate. We suggest that pyruvate productivity could possibly be indirectly improved by increasing specific glucose uptake, for instance by engineering *ptsI* overexpression from a promoter induced in nitrogen‐limited conditions. Ideally, elevated glucose consumption should only occur during the nitrogen‐limited phase as *E. coli* MG1655 pdCas9 psgRNA_pdhR_329 did not benefit from its high glucose consumption rates during the growth phase. In general, glucose consumption rates at the level of this strain are unusual in aerobic fermentations but have been reported for strains with *aceE* deletions [5]. Together with the *aceE* expression data from the microbioreactor experiments it appears that repression of *aceE* was very strong in *E. coli* MG1655 pdCas9 psgRNA_pdhR_329 during the exponential phase which might also explain its lower specific growth rate.

The use case and metabolic behavior of our strains are similar to those from the studies of Michalowski et al. [16] and Moxley and Eiteman [17], so direct comparison of our strains with *E. coli* HGT [16] or *E. coli* MEC826 and *E. coli* MEC905 [17] is feasible. During the exponential phase of bioreactor experiments pyruvate yield and biomass specific pyruvate production rate of *E. coli* MG1655 pdCas9 psgRNA_aceE_234_pdhR_329 were comparable to values reported for *E. coli* HGT and *E. coli* MEC826, but lower than the yield of *E. coli* MEC905. In contrast, *E. coli* MG1655 pdCas9 psgRNA_aceE_234_pdhR_329 achieved a higher maximum specific growth rate than these strains [16]. Specific pyruvate productivity of *E. coli* MG1655 pdCas9 psgRNA_aceE_234_pdhR_329 was lower during the second production phase with resting cells compared to *E. coli* HGT which indicates that stronger repression of pyruvate dehydrogenase or higher glucose uptake rates on the level of *E. coli* HGT may be necessary to improve its production phenotype.

A potential advantage of CRISPRi compared to other genetic modifications to lower gene expression or enzymatic activity is its inherent flexibility to switch off or tune metabolic pathways during a process. A promising strategy for regulating access of different metabolic pathways to the pyruvate pool is the addition of dynamic control circuits [58]. Both a circuit based on PdhR and a dynamic CRISPRi silencing strategy have been applied successfully in *Bacillus subtilis* and the principle can likely be transferred to *E. coli* [59, 60]. Integration of a pyruvate‐sensing circuit based on PdhR into an *E. coli* strain producing a pyruvate‐derived product would require initial modifications of the genomic elements of the *pdhR*, *aceE* and *aceF* loci, but would then enable rapid phenotyping of dynamic control strategies by modulated transcription of dCas9 or sgRNAs targeting key genes of competing pathways.

In conclusion, we successfully engineered CRISPRi knockdown strains for the stable production of pyruvate during two‐phase bioreactor fermentations. An important finding is that targeting *aceEp* with multiple sgRNAs was not successful despite sufficient distance between the target sites. Simultaneously repressing *aceEp* and *pdhRp* improved pyruvate accumulation during the exponential phase and was sufficient to enable constant pyruvate production during a nitrogen‐limited phase. Furthermore, targeting *pdhR* alone was sufficient to enable strong pyruvate production during the growth phase and a continued low‐level accumulation in the nitrogen‐limited production phase. Access to strains with different levels of pyruvate production provides a foundation for controlled production of pyruvate and pyruvate‐derived products in *E. coli*, and *E. coli* MG1655 pdCas9 psgRNA_aceE_234_pdhR_329 or *E. coli* MG1655 pdCas9 psgRNA_pdhR_329 may serve as chassis strains in future investigations.

## CONFLICT OF INTEREST

The authors have declared no conflict of interest.

## Supporting information

Supporting Information

## Data Availability

The data that support the findings of this study are available from the corresponding author upon reasonable request.
