# Supplementary material for: CRISPRi enables fast growth followed by stable aerobic pyruvate formation in Escherichia coli without auxotrophy
Source: Eng Life Sci. 2021 Nov 30;22(2):70–84. doi: 10.1002/elsc.202100021 (PMC8811725; doi:10.1002/elsc.202100021)
Supplement: Supplementary file 1 — Supporting Information [file ELSC-22-70-s001.pdf]

Supporting Information S1: Knockdown of *lacZ*

We designed 3 sgRNAs targeting *lacZ* (237, 238 and 239, Suppl. Fig. 1) and used a previously described sgRNA (236) targeting *lacZ* and an empty sgRNA vector (241) as a control for the functionality of the CRISPRi system (Qi et al., 2013). *E. coli* MG1655 was used as the reference strain with high ß-galactosidase activity and *E. coli* DH5α λ pir was used as a control strain lacking any ß-galactosidase activity. We performed ß-galactosidase assays and found that all sgRNAs targeting *lacZ* reduced ß-galactosidase activity by at least one order of magnitude (Suppl. Fig. 2). In all cases significant downregulation compared to the control strain *E. coli* MG1655 was also observed in the absence of inducer which points towards leaky expression of dCas9 from pdCas9. Enzymatic activity was also reduced with an empty sgRNA vector and more pronounced in the presence of anhydrotetracycline which indicates that small side-effects not related to the presence of sgRNA exist. Presumably, the metabolic burden of plasmid replication and dCas9 expression reduces available cellular resources for ß-galactosidase expression. A residual activity remained in all assays except for the ß-galactosidase assays of the *lacZ*$^-$ control strain *E. coli* DH5α λ pir.

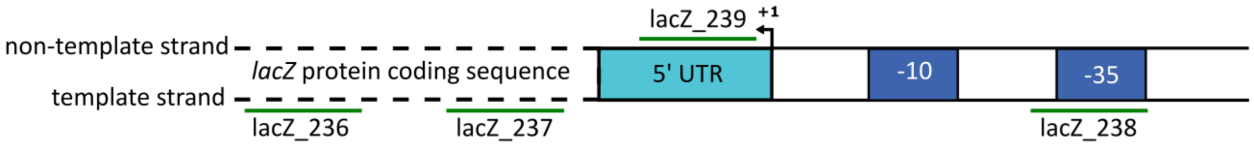

Supporting Figure 1: sgRNA binding sites for CRISPRi mediated knockdown of *lacZ*.

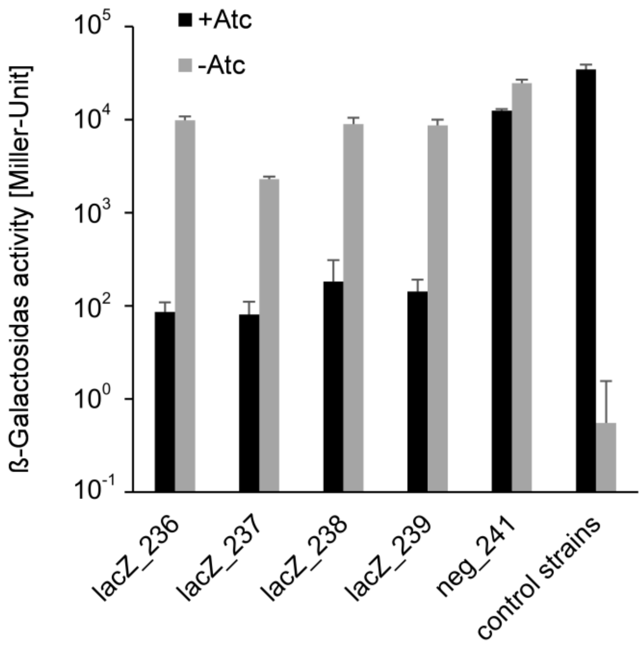

Supporting Figure 2: Knockdown of *lacZ*. ß-galactosidase activity of *E. coli* MG1655 pdCas9 with different psgRNAs targeting *lacZ* (236, 237, 238, 239) or an empty psgRNA (241) with (black bars) or without (grey bars) inducer anhydrotetracycline. *E. coli* MG1655 (black bar) and *E. coli* DH5α λ pir (grey bar) were used as control strains without anhydrotetracycline addition. Error bars indicate SEM (n = 3).

Supporting Information S2: Inducer Concentration

E. coli MG1655 pdCas9 psgRNA_aceE_233 was cultivated in shaking flasks with anhydrotetracycline concentrations ranging from 0.01 µg/ml to 1.0 µg/ml (Suppl. Fig. 2). Anhydrotetracycline serves as the inducer of dCas9 expression from pdCas9. A control fermentation with no addition of anhydrotetracycline was conducted to estimate the effects of leaky expression of dCas9. In all other shaking flask experiments and bioreactor cultivation anhydrotetracycline was added to a final concentration of 0.1 µg/ml as described in the main text.

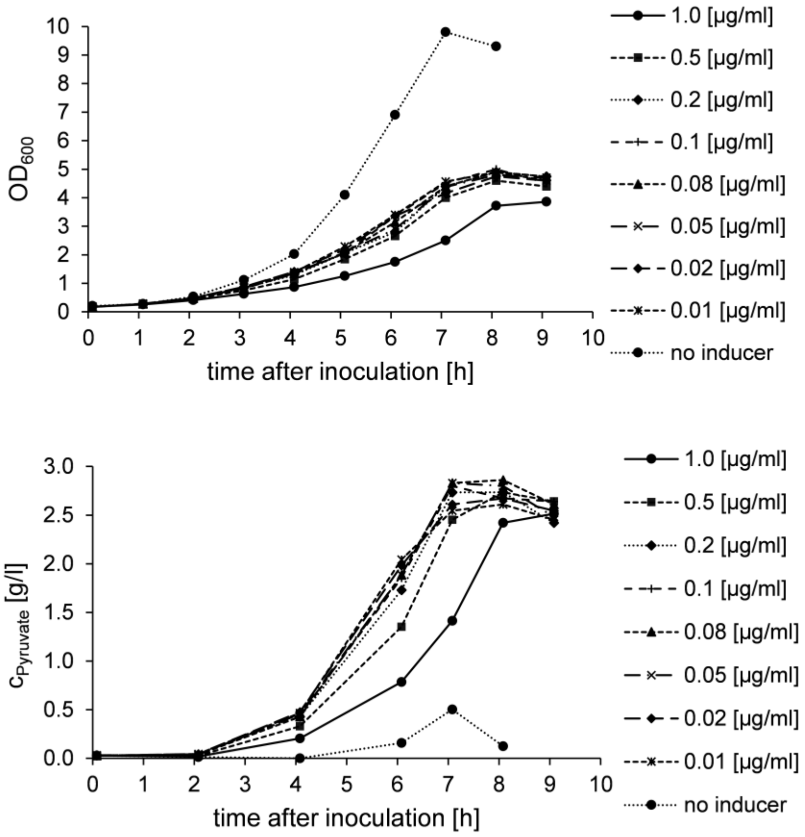

Supporting Figure 3: **Inducer Concentration**. Shaking flask fermentations of *E. coli* MG1655 pdCas9 psgRNA_aceE_233 were conducted with varying addition of anhydrotetracycline as indicated in the figure legend. Data on optical density (upper panel) and pyruvate concentration (lower panel) from single experiments is shown.

Supporting Information S3: Statistical Analysis of qPCR data

Tests were conducted on $\Delta\Delta c_t$-values. One-tailed t-tests were calculated to test the hypotheses that knockdown strains have identical expression levels of *aceE* compared to *E. coli* MG1655 (H_0) or not (H_1). The overall significance level tested was $\alpha = 0.05$. To correct for multiple tests, adjusted significance levels with increased strictness according to Bonferroni-Holm were applied (Holm, 1979).

Supporting Table S1: Results of t-tests for *aceE* expression of strains to reference *E. coli* MG1655

Strain	p-value	Bonferroni-Holm criteria	Accepted hypothesis
**aceE_234_pdhR_329**	1.1073e-05	0.0083	H_1
**aceE_234**	2.2904e-04	0.0100	H_1
**aceE_234_pdhR_327**	0.0011	0.0125	H_1
**pdhR_329**	0.0044	0.0167	H_1
**aceE_232_aceE_234**	0.0103	0.0250	H_1
**aceE_233_aceE_234**	0.0285	0.0500	H_1

A Welch-ANOVA (non-homogeneous variances, Levene: $p = 0.049$) was calculated and showed significant differences between means of the groups: Welch's $F(5, 6.595) = 11.153$, $p = 0.004$. To determine which groups were significantly different from each other a Games-Howell test was calculated for post-hoc analysis of expression differences among the knockdown strains (Games and Howell, 1976). Only strain *E. coli* MG1655 pdCas9 psgRNA_pdhR_329 showed any significant differences to other strains.

Supporting Table S2: P-values of *aceE* expression among knockdown strains (Games-Howell)

Strain A	Strain B	p-value
**pdhR_329**	**aceE_232_aceE_234**	0.118
**pdhR_329**	**aceE_234_pdhR_327**	0.042
**pdhR_329**	**aceE_234**	0.015
**pdhR_329**	**aceE_233_aceE_234**	0.039
**pdhR_329**	**aceE_234_pdhR_329**	0.050

All other strains were not significantly different to each other (data not shown).

References

Games, P.A., Howell, J.F., 1976. Pairwise Multiple Comparison Procedures with Unequal N's and/or Variances: A Monte Carlo Study. Journal of Educational Statistics 1, 113. https://doi.org/10.2307/1164979.

Holm, S., 1979. A Simple Sequentially Rejective Multiple Test Procedure. Scandinavian Journal of Statistics 6, 65–70.

Qi, L.S., Larson, M.H., Gilbert, L.A., Doudna, J.A., Weissman, J.S., Arkin, A.P., Lim, W.A., 2013. Repurposing CRISPR as an RNA-guided platform for sequence-specific control of gene expression. Cell 152, 1173–1183. https://doi.org/10.1016/j.cell.2013.02.022.
